# Amoebic liver abscess in the medical emergency of a North Indian hospital

**DOI:** 10.1186/1756-0500-3-21

**Published:** 2010-01-25

**Authors:** Navneet Sharma, Aman Sharma, Subhash Varma, Anupam Lal, Virendra Singh

**Affiliations:** 1 The Department of Internal Medicine, Postgraduate Institute of Medical Education and Research, Chandigarh-160012, India; 2 The Department of Radiology, Postgraduate Institute of Medical Education and Research, Chandigarh-160012, India; 3 The Department of Hepatology, Postgraduate Institute of Medical Education and Research, Chandigarh-160012, India

## Abstract

**Background:**

Amoebic Liver abscess although fairly common in developing countries, yet, there is limited data on the clinical presentation to the emergency department. A retrospective analysis of 86 indoor cases of Amoebic Liver Abscess presenting to the emergency department over a 5-year period was carried out.

**Findings:**

The mean age of patients was 40.5 ± 2.1 years (male-female ratio = 7:1). Fever, pain abdomen and diarrhea were seen in 94%, 90% and 10.5% respectively. Duration of symptoms less than 2 weeks was seen in 48% cases. Hepatomegaly was present in 16% cases only, a right sided pleural effusion in 14% cases and ascites in 5.7%. On ultrasound, a right lobe abscess was seen in 65%, a left lobe abscess in 13% and multiple abscesses in both the lobes in 22% cases. Seventy one cases underwent per-cutaneous pigtail catheter drainage for a mean period of 13.4 ± 0.8 days. The mortality rate was 5.8%. On multivariate regression and correlation analysis, a higher number of inserted pigtail catheters correlated to mortality.

**Conclusions:**

Amoebic liver abscess presents commonly to the emergency department and should be suspected in persons with prolonged fever and pain abdomen. Conservative management for uncomplicated amoebic liver abscess and insertion of single per-cutaneous pigtail catheter drainage for complicated amoebic liver abscess are efficacious as treatment modalities.

## Introduction

Diseases caused by Entamoeba histolytica manifest as acute infectious diarrhea clinically and pathologically as ulcerative and inflammatory lesion in the caecum and the entire colon [1]. The organism during the invasive stage gains access to the liver via the portal vein where marked tissue destruction occurs resulting in a liver abscess [2-7]. In India, due to poor sanitary condition and a lower socioeconomic status, amoebiasis is endemic and amoebic liver abscess accounts for 3-9% of all cases of amoebiasis [8].

Patients with amoebic liver abscess manifest early with abdominal pain and fever or as fever of unknown origin, weight loss and abdominal pain [7]. Coexisting diarrhea occurs in 30% and it is extremely rare to find amoebic trophozoites in the stool examination [7]. Although, amebic liver abscess occurs mostly in the right lobe, yet, considerable variations exist. In an ultrasonographic analysis of 212 patients of amoebic liver abscess, 16% had multiple abscesses, 35% had left lobe abscess, and in 49% there was a solitary abscess in the right lobe [9]. A recent study from a tertiary care center in North India has shown that polymerase chain reaction is a useful diagnostic tool in demonstrating the culprit organism in pus from amoebic liver abscess [10]. Due to the widespread availability of Ultrasound Imaging at district health care centers, both private and public, emergency departments are places where patients with amoebic liver abscess commonly present. This study was carried out to elucidate the clinical profile of amoebic liver abscess cases admitted in the emergency department.

## Methods

All patients that presented with amoebic liver abscess from January 2000 till December 2004 were included. A retrospective case notes' analysis of all cases of serologically and ultrasonographically confirmed amoebic liver abscess from the medical record library of the institute was carried out. Exclusion criteria were:-

1. The presence of microorganisms other than Entamoeba Histolytica on Gram's stain of pus or on culture.

2. IgM ELISA Serology negative for Entamoeba histolytica

All data regarding the history and general physical examination with special attention to the liver size was recorded. Laboratory investigations noted were complete blood counts, liver function tests, prothrombin time index and a partial thromboplastin time (Kaolin). Data about blood cultures drawn for bacteria and pus drawn during per-cutaneous aspiration was entered. All patients received therapy for liver abscess in form of a combination therapy with either Metronidazole 500 mg 6 hourly intravenously with Cefotaxime 2 grams three times a day parenterally or Metronidazole with Ciprofloxacin 200 mg twice a day parenterally. Metronidazole therapy was given for a period of 10 days whereas antibiotic therapy was continued for three days after the resolution of fever. Indications for per-cutaneous insertion pigtail catheter drainage of amoebic liver abscess were:-

- Failure of medical therapy within 48-72 hours

- Abscess cavity size of > 5 cms with a thin rim (< 1 cm) of liver tissue around it on ultrasound examination

- Left lobe abscess

Pleural fluid, if present, was aspirated and in presence of purulence of the fluid or a reduced pleural fluid sugar <65 mg/dl, a chest tube had been inserted. For ascites, a diagnostic abdominocentesis was performed. The presence of purulence in the ascitic fluid necessitated the insertion of an abdominal catheter for drainage.

For analysis of data, the statistical software SPSS version 10 was used. A multivatiate regression analysis was used to evaluate the relationship of rupture of liver abscess and mortality with various clinical features, ultrasonographic findings and treatment modalities. The Ethics Committee of the institute gave approval for the study.

## Results

In a total of 86 cases, the male-female ratio was 7:1. The mean age of patients was 40.5 ± 2.1 years (range = 13-82 years). The mean duration of fever was 17.9 days (range = 5 - 100 days) and the mean duration of pain was 14.1 ± 1.7 days. Out of 86 patients, 5 (5.8%) died (3 males and 2 females). The duration of symptoms greater than 2 weeks was seen in 52% of cases.

The clinical features of all patients are presented in table 1. Of 86 patients, the liver could not be palpated below the right costal margin in 12.7% (11 cases) and the mean liver span below the right costal margin in the remaining was 3.0 ± 0.15 cms (range = 1-12 cms). A right sided pleural effusion was present in 14% (12 cases), anemia in 62% (53 cases) and leucocytosis in 68% (59 cases). The serum hepatic transaminases levels were raised to greater than 3 times the normal in 35% (30 cases).

**Table 1:** Presenting features of 86 cases of ALA

Features	n (%)
Fever	81 (94)

Pain Abdomen	78 (90.6)

Jaundice	11 (12.7)

Hepatomegaly	13 (16)

Diarrhea	9 (10.5)

Abdominal Distension	3 (3.5)

Cough	2 (3.5)

Tachycardia	14 (12)

Hypotension	2 (2.3)

Deranged Prothrombin time index (<80%)	37 (43)

Hypoalbuminemia (< 3 gm/dL)	41 (47.6)

Alcohol consumption	40 (46.5)

On ultrasound examination, a right lobe liver abscess was seen in 65%, left lobe abscess in 13% and multiple abscesses in both the lobes in 22% cases. Eleven patients had rupture of the abscess; right pleural rupture in 7, peritoneal rupture in 3 and both right pleural and peritoneal rupture in 1 patient respectively (table 2). Seventy one cases underwent per-cutaneous pigtail catheter drainage and the remaining cases were managed conservatively. The mean in-hospital stay of patients was 13.4 ± 0.8 days (range = 2-35 days) and the mean duration of time that the pigtail catheter drainage was carried out was for 12.2 ± 1.45 days (range = 4-28 days). There was no statistically significantly difference in the drainage group (n = 71) as compared to the group managed conservatively (n = 15) with respect to the duration of fever, duration of pain abdomen, jaundice, serum albumin, liver span and prothrombin time index. Of all ALA cases, 9.3% required insertion of a chest tube into the right pleural cavity for draining the ruptured abscess. Table 2 shows the distribution of 86 cases with respect to those that underwent per-cutaneous catheter drainage of liver abscess concomitantly with chest tube drainage.

**Table 2:** Number of Pigtail Catheters Versus Chest Tube Inserted in 86 cases of ALA

Count		Chest Tube Inserted		Total
		**no**	**yes**	

Number of Pigtail Catheter	0	15	0	15
	1	50	5	55
	2	8	3	11
	3	4	0	4
	4	1	0	1
Total		78	8	86

Seventy cases received a combination of Metronidazole and Cefotaxime therapy and 16 cases a combination of Metronidazole and Ciprofloxacin. There was no statistically significant differences in-between the 2 groups. On multivariate regression and correlation analysis, a higher number of inserted pigtail catheters for amoebic liver abscess drainage correlated to mortality (R = 0.53, Pearson's Correlation significant at 0.001). Table 3 shows the cross-tabulation of the number of per-cutaneously inserted pigtail catheters to outcome in our ALA cases. Although, patients with ruptured amoebic liver abscess had a longer in-hospital stay than those managed conservatively, yet, this was not statistically significant.

**Table 3:** Number of Pigtail Catheters versus Clinical Outcome of 86 cases of ALA

Count		Outcome		Total
		**survived**	**Deaths**	

Number of Pigtail Catheter	0	15	0	15
	1	54	1	55
	2	9	2	11
	3	2	2	4
	4	1	0	1
Total		81	5	86

## Discussion

The WHO estimates that Entameoba histolytica causes 50 million cases and 100,000 deaths annually, making this disease the second leading cause of death from protozoal diseases [1-4]. Although infection with Entamoeba histolytica occurs world-wide, yet, liver abscess is the most common extraintestinal complication in 3% to 9% of patients [1-8]. Diagnosis of amoebic liver abscess is usually straightforward on the basis of the clinical, epidemiological, serological and ultrasonographic findings.

Amoebic liver abscess arises from the hematogenous spread of the trophozoites of Entameba histolytica from the intestinal mucosa to the liver through the portal vein. The disease is suspected in endemic areas in persons presenting with fever, pain abdomen and liver tenderness [7,11,12]. Compared to pyogenic liver abscesses, patients with amoebic abscesses are often younger, more acutely ill with fever and right upper quadrant pain, and are usually from high prevalence areas [11,12]. The mean age of our patients with amoebic liver abscess was 40 years and was comparable to other studies [11]. The frequency of fever and pain abdomen is 67-87% and 62-94% of patients with amoebic liver abscess respectively in different series [10]. In our study, these two symptoms of fever and pain abdomen occurred in 94 and 90% respectively [10]. From India, Sharma et al in a study of 70 cases of amoebic liver abscess found hepatomegaly in 84%, pleural effusion in 10% and ascites in 4% cases [8]. In contrast, hepatomegaly (16%) was not a predominant feature of amoebic liver abscess in our study. Forty six percent cases in our study were consuming alcohol and this may account for the lesser occurrence of hepatomegaly. In our study, pleural effusion was seen in 14% and ascites in 5.7% cases respectively.

From India, earlier series showed jaundice in 45%-50% of cases of amoebic liver abscess, but, after the advent of invasive catheter drainage, coupled with effective anti-amoebic therapy, it has become less common [11-13]. Jaundice occurred in 12.7% cases in our study. The pathophysiology of jaundice remains controversial and various explanations of jaundice are; pressure of abscess cavity on hepatic ducts [14-17] and cholestasis [18-20]. Recently, in 12 cases of amoebic liver abscess with jaundice, the formation of a bilio-vascular fistula was seen [21]. Duration of symptoms longer than 2 weeks is seen in 14-41% in different series [11]. In a study of amoebic liver abscess by Amarapurkar and colleagues of 131 patients, the duration of symptoms less than 2 weeks was seen in 83.9% of cases [22]. In this study, 84% presented within 2 weeks and mild elevations of serum transaminases was seen in 19.8% cases. In our study, duration of symptoms less than 2 weeks was evident in 48% cases and raised liver enzymes more than 3 times the normal occurred in 35% cases. The lesser rise of serum transaminases seen in the study by Amarapurkar and colleagues could have been due to an earlier detection of ALA on abdominal ultrasonography [22]. In our study, diarrhea occurred in 10.5% and cough in 3.5% cases whereas in other studies, these 2 symptoms occur in 14-40% and 8-24% cases respectively [11].

Abdominal Ultrasound is the gold standard for diagnosing liver abscesses. Sonographically, in ALA, 4%-42% cases have multiple abscesses, 20%-35% have an abscess in the left lobe, and the remaining 49%-80% have a solitary abscess in the right lobe [9,13]. Our study showed multiple abscesses in 22% cases, a solitary left lobe abscess in 13% cases and a single right lobe abscess in 65% of cases.

Atelectasis and pleural effusions are common complications of ALA. Pleural effusions occur mostly frequently in the right lobe and cause cough and chest pain. Respiratory distress can follow as a sequel to ALA rupturing through the diaphragm. Such a course has been shown to unravel in 7-20% of cases of ALA and in this study accounted for 14% of cases [11-13]. Of all our patients, 9.3% required a chest tube insertion for pleural drainage. In 2-7% of cases of ALA, a peritoneal rupture can cause shock and peritonitis [23-25]. A peritoneal rupture occurred in 4 cases, of which there was 1 case with both right pleural and peritoneal rupture. In recent years, with the advent of pigtail catheter drainage, the role of surgical exploration in ALA ruptured into the peritoneal cavity has been mainly confined to haemodynamically unstable patients [26]. None of our patients underwent surgical exploration. Although, the in hospital stay of patients of ruptured ALA was more than those managed conservatively, yet, it did not reach statistical significance. This may be due the small size of the group of patients managed conservatively.

The overall mortality rate seen in ALA from various series ranges from 2-15% [11]. In our study, the mortality rate was 5.8% and correlated to an increased number of pigtails catheters that were inserted in the ALA cavity. This fact is of particular concern and awaits further confirmation across a larger study.

## Conclusion

In India, amoebic liver abscess commonly presents to the emergency department. Amoebic liver abscess should be suspected in persons presenting with prolonged fever and pain abdomen to the emergency department and hepatomegaly as the presenting feature is not always present. Conservative management for uncomplicated amoebic liver abscess and insertion of single per-cutaneous pigtail catheter drainage for complicated amoebic liver abscess are both efficacious as treatment modalities.

## Competing interests

The authors declare that they have no competing interests.

## Authors' contributions

NS-Associate Professor in collecting data, writing, design, statistical calculation and preparing manuscript; AS-Assistant Professor in collecting & recording data; SV-Professor helped to prepare the study design; AL-Assistant Professor in helping with radiology & VS-Additional Professor in helping with methods and discussion. All authors read and approved the final manuscript.
